# Endoscopic Ventriculocysternostomy, Magendie Foraminoplasty, and Plexusectomy With Craniovertebral Shunt Placement in a Pediatric Patient With Hydrocephalus and VACTERL Association: A Novel Treatment Option

**DOI:** 10.7759/cureus.58845

**Published:** 2024-04-23

**Authors:** Ruslan I Asadov, Edwin Bernard, Brenda Enelis

**Affiliations:** 1 Neurological Surgery, Scientific and Practical Center of Specialized Medical Care for Children Named After V.F. Voino-Yasenetsky of the Health Department of the City of Moscow, Moscow, RUS; 2 Neurosurgery, Russian University People’s Friendship (RUDN) European Medical Centre (EMC) Medical School, Moscow, RUS

**Keywords:** craniovertebral shunt, vaterl association, pediatric neurosurgery, pediatric hydrocephalus, plasty of the magendie foramen, ventriculocysternostomy

## Abstract

Endoscopic third ventriculocysternostomy (ETV) is a minimally invasive neurosurgical technique with good results in the treatment of obstructive hydrocephalus. The VACTERL (vertebrae, anorectal, cardiovascular, tracheal, esophageal, renal, limb defects) association, or VATER syndrome, is defined as congenital malformations, mostly derived from the mesoderm, affecting specific areas. It is diagnosed by the presence of at least three of the seven characteristic malformations that describe it. The association of this pathology and obstructive hydrocephalus in pediatric age is not common, making management and conventional neurosurgical procedures difficult due to the number of underlying pathologies. In this study, we report the management of hydrocephalus and VACTERL association with multiple congenital malformations in a 30-day-old premature neonate (birth at 29 weeks). Operations performed prior to admission to our service included: coloesophagoplasty and placement of esophagostoma in the left anterior cervical region, perineal anorectoplasty, gastrostomy and placement of sigmoidostomy in the left anterior abdominal wall, relaparotomy, gastric suture, sanitation, and abdominal drainage. Upon admission, the patient showed a Grade 3 intraventricular hemorrhage and internal occlusive hydrocephalus due to circulatory blockage of the cerebrospinal fluid (CSF) at the level of the outlet of the fourth ventricle. This was accompanied by intracranial hypertension and refractory cervical syringomyelia. We performed endoscopic ventriculocysternostomy plus plexusectomy plus Magendie foraminoplasty with craniovertebral shunt placement, achieving excellent results after two interventions. This is the first case described in the literature placing a craniovertebral shunt using a lateral-ventricle-to-the-subarachnoid-spinal-space-stenting technique in a patient with VACTERL association, which represents an innovation in the field of minimally invasive pediatric neurosurgery.

## Introduction

The endoscopic third ventriculocysternostomy (ETV) is a minimally invasive surgical intervention performed to treat obstructive hydrocephalus. The goal of ETV is to establish communication between the third ventricle and the interpeduncular cistern [1,2].

The ETV is a simple anatomical and physiologic alternative to treat many types of hydrocephalus without significant morbidity and comprises contemporary concepts of minimally invasive neurosurgery. It consists of a supraciliary incision, a small craniotomy, and fenestration of the lamina terminalis and Liliequist membrane [1]. The failure pattern for ETV is different from shunting, with a higher early failure rate but improved long-term failure-free survival rates. There is insufficient evidence to recommend the use of ETV in premature infants with posthemorrhagic hydrocephalus [3,4,5].

The VACTERL association (VATER syndrome), is defined as congenital malformations that mostly derive from the mesoderm, affecting specific areas. The acronym in English is V: vertebral anomalies, A: anal atresia, C: cardiovascular anomalies, TE: tracheo-esophageal fistula, R: renal anomalies, L: limb defects). It is diagnosed by the presence of at least three of the previously mentioned malformations. Early diagnosis in the prenatal stage with ultrasound is important in order to perform an early approach and reduce postsurgical morbidity and mortality. The most common feature is a vertebral anomaly, found in 60%-80% of cases. Tracheo-esophageal fistula is seen in 50%-80% of cases and renal malformations in 30% of patients. Limb defects, including thumb aplasia/hypoplasia, polydactyly, and radial agenesis/hypoplasia, are present in 40%-50% of cases. Anorectal defects, such as imperforate anus/anal atresia, are challenging to detect prenatally [6,7,8].

The etiology of the VACTERL association remains unclear and probably has a multifactorial origin, so it cannot be classified as a specific syndrome, furthermore, its components are variable. It has been suggested that exposure to estrogens, progestins, or both during the first trimester of pregnancy may be a cause of this entity. Its occurrence is sporadic although the appearance of several cases in a family suggests an inheritance of an autosomal nature, as are cases of VACTERL with hydrocephalus, which follow a pattern of autosomal recessive inheritance [9]. A new discovery indicates that a copy number variation (CNV) contributes to VACTERL pathogenicity, identifying a novel CNV in 8p23 and 12q23.1 in a case of anorectal malformations (ARMs)-related VACTERL association [10]. DNA methylation disturbances in the ovum are a likely cause, with epigenetic links to individual components and to folate effects before conception, explaining diverse fetal and placental findings and providing a link to fetal origin hypothesis-related effects [11].

The presence of this pathology, simultaneously associated with obstructive ventricular hydrocephalus, results in difficult surgical management because it is a multidisciplinary surgery that involves several specialties in which therapeutic options are scarce due to the number of underlying pathologies. This is the first case described in the literature regarding placing a cranio-vertebral shunt using a lateral-ventricle-to-a-subaracnoid-spinal-space stenting technique in a premature patient with VATERL association.

Therefore, we present a rare case of a 30-day-old neonate with a history of premature birth (29 weeks) and VACTERL association related to multiple congenital malformations: atrial septal defect (4.5 mm), esophageal atresia, and anal atresia (rectoperineal fistula). Procedures performed prior to admission to our department were coloesophagoplasty, esophagostoma in the left anterior cervical region, perineal anorectoplasty, placement of a sigmoidostomy in the left anterior abdominal wall, relaparotomy, gastric suture with sanitation, and placement of abdominal drainage. We diagnosed a Grade 3 intraventricular hemorrhage and internal occlusive hydrocephalus due to blockage of the circulation of the cerebrospinal fluid (CSF) at the level of the fourth ventricle, accompanied by intracranial hypertension and secondary cervical syringohydromyelia.

## Case presentation

**Figure 1:**
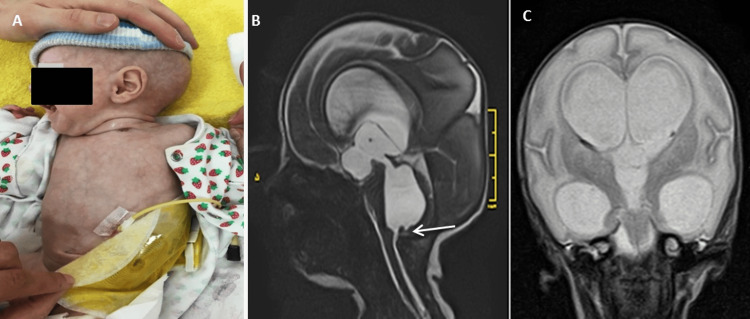
Patient images. A - The patient's condition at the moment of admission to our department (head with hydrocephalic shape, an esophagostomy in the lower left side of the neck, and the colostomy bag placed on the left abdominal wall); B - Sagittal view showing occlusion of the cerebrospinal fluid pathways at the level of the outlet sections of the 4th ventricle (arrow); C - Coronal view showing a considerable ventriculomegaly at the level of all ventricles with signs of intraventricular hemorrhage.

A 30-day-old male patient was brought to our pediatric neurosurgery service with a history of premature birth at 29 weeks, birth weight of 1450 grams, progressive increase in head circumference of 32 cm, bulging of the major fontanelle of 4 cm x 4 cm, and irritability. The patient had a history of multiple congenital malformations: VACTERL syndrome associated with atrial septal defect (4.5 mm), esophageal atresia, and anal atresia (rectoperineal fistula). The operations performed included removal of the esophagus and placement of esophagostoma in the left anterior cervical region, gastrostomy, placement of a sigmoidostomy in the left anterior abdominal wall, relaparotomy, gastric suture, sanitation, and abdominal drainage. Cranial and cervical magnetic resonance imaging (MRI) reflected an abnormal expansion of the lateral ventricles and the third ventricle with the presence of interior blood clots. The fourth ventricle was dilated, accompanied by lateral eversion and bulging, causing cerebral hypertension. The Luschka and Magendie foramina were occluded due to the presence of abnormal subarachnoid adhesions, and the spinal cord presented refractory syringomyelia at the cervical level. These factors led to a diagnosis of Grade 3 spontaneous intraventricular hemorrhage and posthemorrhagic progressive occlusive hydrocephalus due to blockage in the outlet of the fourth ventricle (Figure 1). Cerebrospinal fluid (CSF) examination showed cell counts - 30-35x10⁶/l (leukocytes - 6/field, neutrophils - 18/field, monocytes - 6/field, protein - 0.8 g/l, erythrocytes - 10-15/field). Therefore, after discussing the case with our multidisciplinary team, we decided on surgical intervention. We performed an ETV plus plexusectomy plus Magendie foraminoplasty with a craniovertebral shunt placement.

Surgical procedure

Under general anesthesia and endotracheal intubation, the patient was placed in a supine position. The head was in a neutral position and elevated to 30 degrees. After asepsis and antisepsis, we marked 2.5 cm laterally from the sagittal suture and 1.0 cm anterior to the coronal suture (Kocher point). We made an arcuate incision over the soft tissue in the right frontal paramedial region. The fontanelle membrane was exposed and a longitudinal incision was made over the fontanelle membrane and dura mater (DM). Then it was electrocoagulated. Subsequently, we began irrigation with normal saline solution (0.9%) through a guided cannula. We guided the endoscope through the foramen of Monro where it traversed until it reached the third ventricle. We observed that the bottom of the third ventricle was thin and bent downward. With the aid of a monopolar wire coagulator, we created a 5-mm-diameter hole in the lower part of the third ventricle, where the diencephalic sheet of the Liliequist membrane was perforated.

The endoscope was inserted as far as the cisterns allowed, where the arachnoid adhesions were dissected. With a single incision, we used a rigid endoscope with extremely careful internal movements and very little amplitude. The endoscope was moved transferentially to the posterior parts of the third ventricle and placed over the opening of the cerebral aqueduct while the CSF expanded dramatically. The endoscope was moved through the aqueduct of Silvius to the fourth ventricle mounted over the projection of the foramen of Magendie, where we could see a complete occlusion of the exit orifice of the fourth ventricle blocking the flow of CSF. In the region of Magendie, we identified a thin planar spike and dissected it using a monopolar electrode. We then placed a Medtronic (Minneapolis, USA) ultra-small diameter (2.1 mm diameter) silicone ventricular catheter (stent), pierced along the cavity of the anterior horn of the right lateral ventricle and the third ventricle. In this way, the CSF was carried through the tubing from the brain to the fourth ventricle and then through the opening formed in the membrane area of the foramen of Magendie, where the catheter was inserted into the posterior spinal subarachnoid space.

We brought the endoscope to the right lateral ventricle, and the choroid plexus was coagulated throughout its entire length. After coagulating the glomus zone of the choroid plexus, it was possible to partially cut the rest of the choroid plexus. The hypertrophied choroidal glomal portion, once coagulated, was fixed at the limbus in the coagulated vessels. In addition, the endoscope moved through the interventricular septal defect. We repeated the process in the left lateral ventricle, where we performed coagulation and partial removal of the choroid plexus in the manner described above. We gradually withdrew the endoscope from the ventricles. The ventricular catheter was fixed to the periosteum by a nodular suture behind the cuff. The total length of the intracranial segment of the ventricular catheter was 14 cm. We placed the distal end of the catheter in the subaponeurotic space of the right parietal region. We sutured the DM. We placed a TachoComb (Nycomed Pharma, Zurich, Switzerland) plate (hemostatic and sealing material) on the DM seam line. We layered sutures into the wound and applied sterile dressings and bandages. The patient stayed in the ICU with endotracheal oxygenation therapy for the first 24 hours, the tube was removed and an oxygen cannula was placed for the next 3 days, and on the fourth day, he went to a hospital room for daily wound care. After 8 days the sutures were removed. The patient was discharged with corresponding guidelines for the parents. The patient attended monthly follow-ups in neurology to monitor his neurological status and measure the cranial perimeter. This was coordinated through our department once a month.

**Figure 2:**
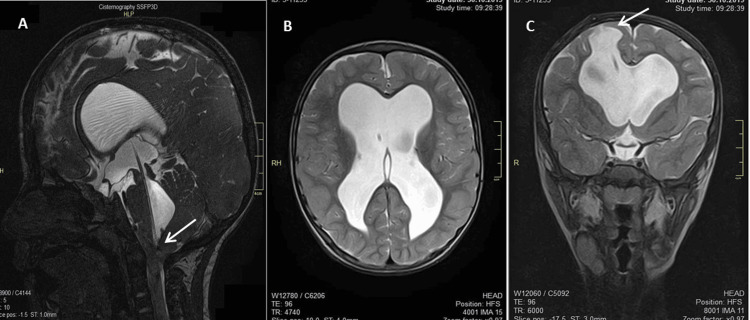
Magnetic resonance sequence on T2 (31 months after first intervention). A - Sagittal view calculating the approach and the level at which the catheter is occluded causing malfunction (C3 vertebrae); B - coronal and C - axial views showing a moderate abnormal expansion of the lateral ventricles and the third ventricle. In the course of the catheter at the level of the right frontal region, a defect due to post-surgical changes can be seen (arrow).

After 2 years, the infant returned to our service due to constant headaches accompanied by irritability and hypoactivity. A CT scan revealed moderate expansion of all parts of the ventricular system, including the fourth ventricle, indicating that the drain installed in the posterior subarachnoid space was occluded at the level of the C3 vertebra. The cerebral and subarachnoid convexity spaces were traceable and not expanded, with no midline shift. We detected no destructive traumatic bone changes, internal occlusive hydrocephalus due to shunt dysfunction, or intraventricular hemorrhage. An MRI showed dislocation of the middle structures and thinning of the cerebral septum and the corpus callosum. We observed a defect in the course of the catheter in the right frontal region (Figure 2). After a careful radiological revision with the neurologist and radiologist, we reached several conclusions, diagnosing intraventricular hemorrhage, internal occlusive hydrocephalus (blockage at the outlet of the fourth ventricle), adhesions after stent placement, negative dynamics of the CSF at the level of the craniocervical transition, and stent failure. We admitted the patient for surgical reintervention.

Surgical re-intervention

The patient was placed under general anesthesia and endotracheal intubation in a supine position. His head was in a neutral position and elevated to 30 degrees. After asepsis and antisepsis, marking was performed 2.5 cm laterally from the sagittal suture and 1.0 cm anterior to the coronal suture (Kocher's point), and 2.5 cm laterally from the sagittal suture and 1.0 cm in front of the coronary suture. We made an arcuate soft tissue incision in the frontal region on the right along the old postoperative scar, exposing the frontal bone on the right, the existing burr hole in the bone, and the previously installed stent (ventricular catheter). We inserted an endoscope along the length of the stent into the anterior horn cavity of the lateral ventricle, and the endoscope continued through the right foramen of Monro into the third ventricle cavity. We observed the floor of the third ventricle, which was thickened, deformed, and bent downwards and the third ventriculostoma left during the previous surgery. The hole was obliterated by a thin arachnoid membrane. With the aid of a monopolar coagulator, we reshaped a 5-mm-diameter hole in the lower part of the third ventricle. The endoscope was inserted into the interstitial cistern, where we dissected the arachnoid adhesions. We then brought the endoscope to the posterior part of the third ventricle over the cerebral midbrain aqueduct where the CSF supply was dramatically expanded.

**Figure 3:**
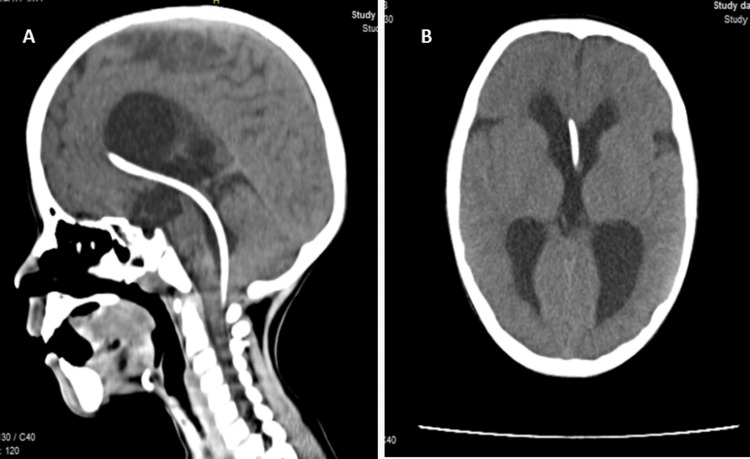
Control CT scan (four weeks after reintervention). A - Sagittal view showing the catheter’s trajectory, proximally from the right lateral ventricle and placed distally into the spinal subarachnoid space; B - Axial view shows the proximal catheter from the right lateral ventricle passing through the foramen of Monro to the third ventricle.

**Figure 4:**
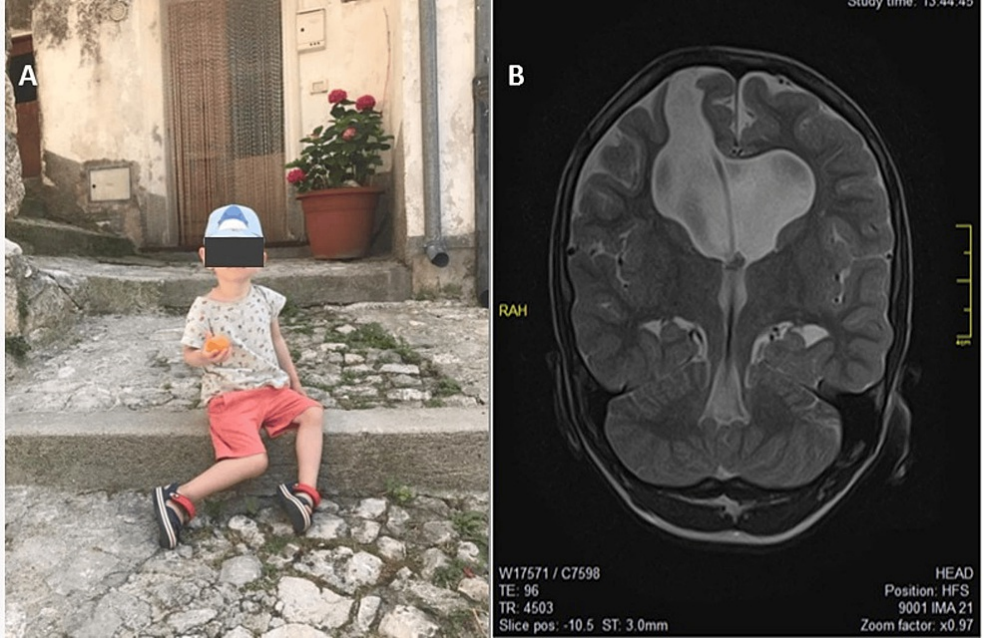
Control studies images showing the efficacy of surgical management. A - The child is having a normal life after all; B - MRI coronal cut T2 window showing the trajectory of the catheter and how it is perfectly working.

**Figure 5:**
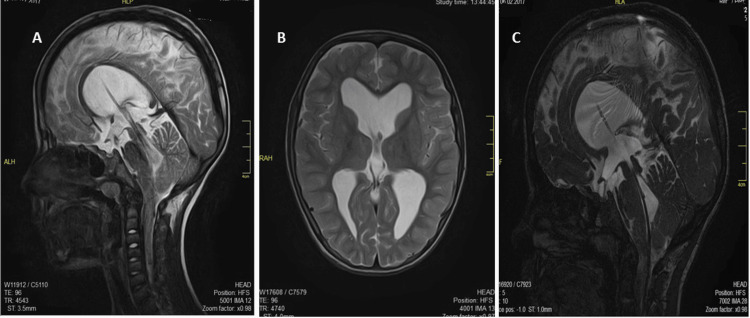
Post-surgical control at five years of age. A - MRI sagittal view showing the catheter from the lateral ventricle through the foramen of Monro, third ventricle, Silvius aqueduct, and fourth ventricle to the spinal subarachnoid space; B - Axial view showing the catheter passing through the Monro to the third ventricle; C - Cisternography, shows the drainage installed from the lateral ventricles to the posterior subarachnoid space at the level of the C5 vertebra.

We guided the endoscope through the foramen of Monro to the limits of the fourth ventricle mounted over the projection of the opening of Magendie. We observed that the tip of the ventricular catheter completely obstructed the opening of the foramen of Magendie. All the catheter openings were located above the plane of the Magendie opening. The foramen of Magendie itself was passable, and the orifice was somehow enlarged. We inserted the available catheter through the Magendie to about 2.0-2.5 cm. The perforated end of the catheter was thus in the area of ​​​​the cisterna magna, and the occlusion of the Magendie orifice was eliminated (Figure 3). The endoscope was gradually withdrawn from the ventricles. The ventricular catheter was fixed to the periosteum with a nodular suture behind the angle clip. The distal end of the catheter was placed in the subaponeurotic space of the right parietal region. We sutured the DM and placed a TachoComb plate on the DM seam line. We layered sutures in the wound and applied aseptic bandages. Postsurgical extubation and management ensued in the intensive care unit for 24 hours with antibiotic therapies (ampicillin, amikacin), infusion therapy, hemostatic therapy, vikasol, and dopamine (use S.O.S.). After the observation period, the patient went to the common room for general care and was discharged 10 days after his reoperation. We conducted control studies showing the efficacy of surgical management (Figures 4, 5). Prior to discharge orientations were given to the parents about home care and periodic assistance from neurology and neurosurgery with monthly appointments.

Results

The patient recovered in a healthy manner. He currently goes to school and integrates into daily activities. The patient leads a normal life. Sometimes it is a little difficult for him to memorize some things. He likes to break dance (but without head tricks), swim (he is one of the best in his group), ride his bike, study English, and go to school. In general, he has good neurocognitive development thanks to the fact that he studies and trains a lot, receives all the support and cooperation of his parents, and is very active. The child has an excellent quality of life (Figures 4, 5).

## Discussion

The VACTERL association or VATER syndrome is a rare condition and is associated with a high rate of morbidity and mortality. This association is characterized by the presence of various malformations in various combinations; in recent studies, it is classified as an association and not as a syndrome, with multifactorial etiology. Therefore, it is necessary to make the diagnosis prenatally with ultrasound identification of these anomalies. The range of morbidity and mortality depends on the complications of the postoperative period and renal failure, which may occur in these patients [8,9,10,11].

The association of this disease with hydrocephalus in newborns has been present in some patients, and its treatment creates a serious neurosurgical challenge. To our knowledge, there are currently no studies that examine the subject in depth, which renders neurosurgical and therapeutic treatment options difficult because of the multiple underlying pathologies that form this complex association of diseases. For this reason, and after an in-depth multidisciplinary analysis, we decided that the best alternative was to perform an endoscopic ventriculocysternostomy plus plexusectomy plus Magendie foraminoplasty with craniovertebral shunt placement. The obtained results confirm that this is an excellent surgical option without further compromising the physiological and physical condition of the patient. Craniovertebral stenting is mostly used in pediatric patients with refractory or recurrent syringomyelia, and the technique usually used is fourth-ventricle-to-spinal-subarachnoid- space stenting [12,13]. This is the first case described in the literature placing a craniovertebral shunt using a stenting technique from the lateral ventricle to the spinal subarachnoid space in a patient with VACTERL association.

Despite the complexity of this clinical case, we obtained excellent results. The external ventricle drainage (EVD) was not necessary during the first intervention, since an active ventricular lavage was performed, leaving the ventricles completely permable as it is described in the surgical procedure. The cause of the second surgery was a spontaneous intraventricular hemorrhage, and stent occlusion at the level of the C3 vertebra.

A reintervention was necessary due to complications that are common in any other standard shunting technique. To date, this surgical technique has not yet been described in the literature to treat this kind of case; in other cases, the surgical indication was based on the presence of refractory syringomyelia in patients already treated for Chiari malformation or in patients who developed scarring at the level of the outlets of the fourth ventricle following posterior fossa tumor surgery [14].

This technique proves to be effective. The patient is currently stable, lives a normal life, and can carry out his daily activities like any child of his age. Given these results and with the clarification that more studies and case experiences will be needed in the future, we consider with no doubt that the technique is a very good therapeutic option for these kinds of patients.

## Conclusions

The endoscopic ventriculocysternostomy plus Magendie foraminoplasty and plexusectomy with craniovertebral shunt placement is a novel therapeutic option in pediatric patients with hydrocephalus and VACTERL association with contraindications to traditional shunting techniques. After performing this surgical technique, it has been proven that it is possible to restore a dynamic communication of the CSF, which is perfectly reabsorbed at the spinal level. This new therapeutic option improves the survival rate and quality of life for these patients with a similar situation. Early diagnosis in the prenatal stage with ultrasound is important in order to perform an early approach and reduce postsurgical morbidity and mortality. The technique described in this article has been performed for the first time in history in a patient with this condition, demonstrating excellent results, which represents an innovation in the field of minimally invasive pediatric neurosurgery.
